# Supplementary figures and images for: I-FABP is decreased in COVID-19 patients, independently of the prognosis
Source: PLoS One. 2021 Apr 15;16(4):e0249799. doi: 10.1371/journal.pone.0249799 (PMC8049236; doi:10.1371/journal.pone.0249799)

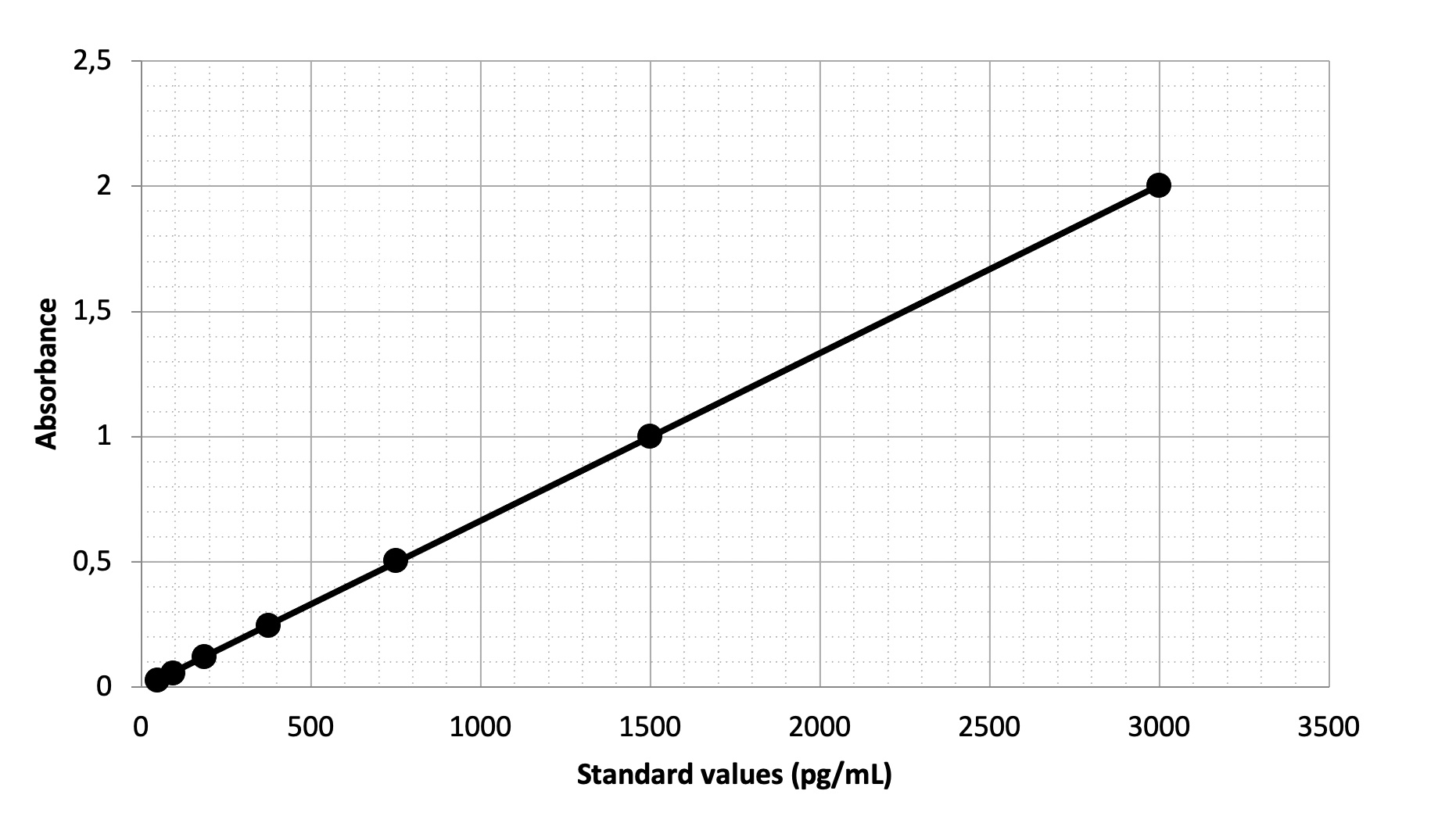

Supplement: S1 Fig — Hycult ELISA assay was used as recommended by the manufacturer. (TIF) [file pone.0249799.s001.tif]

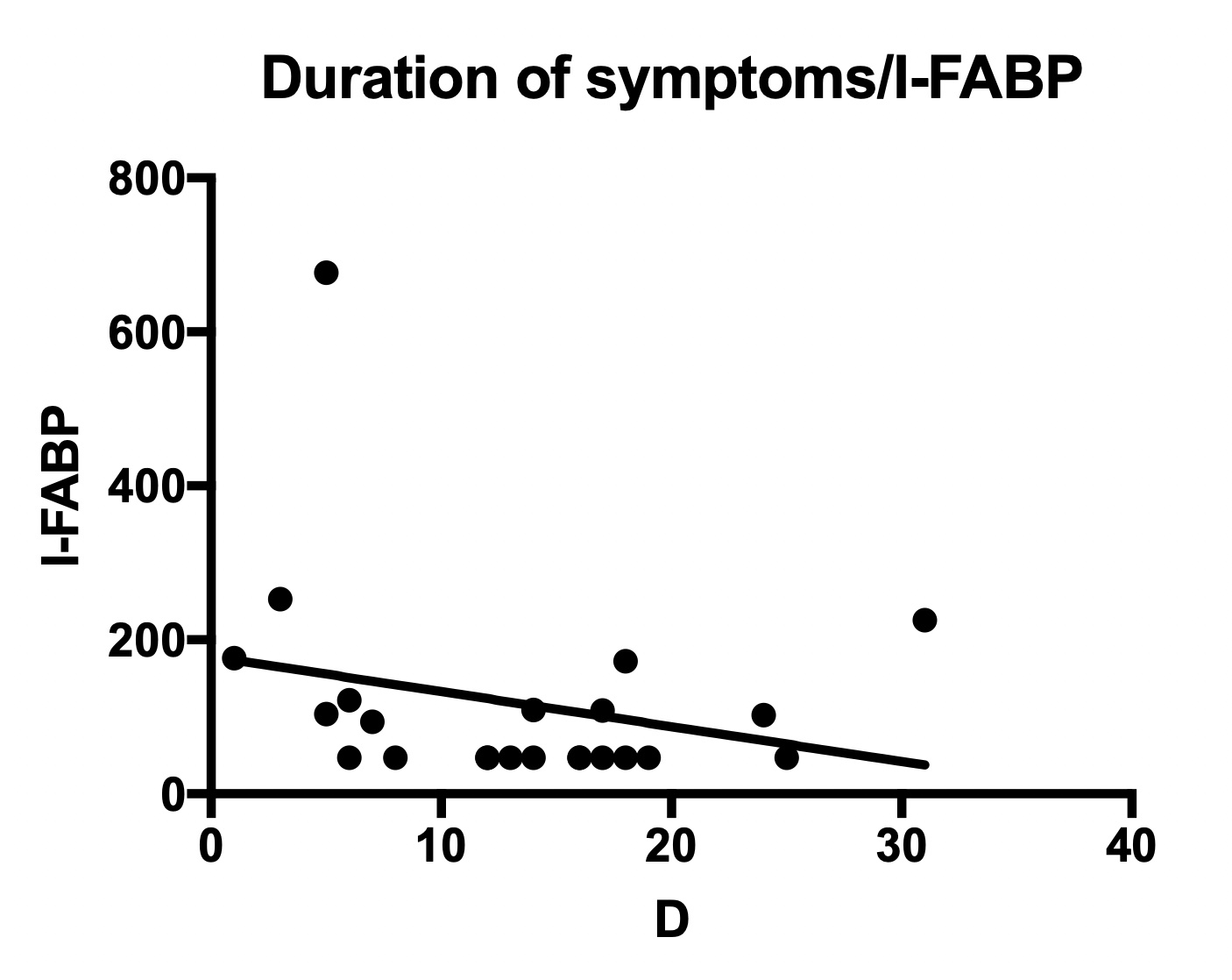

Supplement: S2 Fig — No significant association was seen (R2 = 0.06327). (TIF) [file pone.0249799.s002.tif]
