# Supplementary material for: I-FABP is decreased in COVID-19 patients, independently of the prognosis
Source: PLoS One. 2021 Apr 15;16(4):e0249799. doi: 10.1371/journal.pone.0249799 (PMC8049236; doi:10.1371/journal.pone.0249799)
Supplement: S1 Table — (DOCX) [file pone.0249799.s004.docx]

**S1 Table.**

|  | I-FABP |
| --- | --- |
| Age | 0.0936 |
| BMI | 0.4621 |
| Respiratory rate | 0.9381 |
| Heart rate | 0.3794 |
| Leukocytes | 1.3 10^-6^ |
| Hemoglobin | 0.9660 |
| Platelets | 0.6857 |
| PMN | 3.2 10^-6^ |
| Lymphocytes | 0.01829 |
| Fibrinogen | 0.63915 |
| D-Dimer | 0.58752 |
| CRP | 0.67376 |
| AST | 0.40363 |
| ALT | 0.69774 |
| LDH | 0.80986 |
| Ferritin | 0.21715 |
| Troponin | 0.62840 |
| Citrulline | 0.180532 |

BNP: brain natriuretic peptide; AST: ASAT; ALT: ALAT; BMI: Body mass index; CRP: C reactive protein; LDH: lactate dehydrogenase.
